# A comparison of breast and lung doses from chest CT scans using organ‐based tube current modulation (OBTCM) vs. Automatic tube current modulation (ATCM)

**DOI:** 10.1002/acm2.13198

**Published:** 2021-05-03

**Authors:** Rick R. Layman, Anthony J. Hardy, Hyun J. Kim, Ei Ne Chou, Maryam Bostani, Chris Cagnon, Dianna Cody, Michael McNitt‐Gray

**Affiliations:** ^1^ Department of Imaging Physics The University of Texas MD Anderson Cancer Center Houston TX USA; ^2^ Materials Engineering Division/Non‐destructive Evaluation Group Livermore National Laboratory Livermore CA 94550 USA; ^3^ Department of Radiological Sciences David Geffen School of Medicine University of California, Los Angeles Los Angeles CA USA; ^4^ Fielding School of Public Health University of California Los Angeles Los Angeles CA USA; ^5^ Physics and Biology in Medicine Graduate Program David Geffen School of Medicine University of California Los Angeles Los Angeles CA 90024 USA

**Keywords:** breast and lung dose, organ‐based modulation, tube current modulation

## Abstract

**Purpose:**

The purpose of this work was to estimate and compare breast and lung doses of chest CT scans using organ‐based tube current modulation (OBTCM) to those from conventional, attenuation‐based automatic tube current modulation (ATCM) across a range of patient sizes.

**Methods:**

Thirty‐four patients (17 females, 17 males) who underwent clinically indicated CT chest/abdomen/pelvis (CAP) examinations employing OBTCM were collected from two multi‐detector row CT scanners. Patient size metric was assessed as water equivalent diameter (*D_w_*) taken at the center of the scan volume. Breast and lung tissues were segmented from patient image data to create voxelized models for use in a Monte Carlo transport code. The OBTCM schemes for the chest portion were extracted from the raw projection data. ATCM schemes were estimated using a recently developed method. Breast and lung doses for each TCM scenario were estimated for each patient model. CTDI_vol_‐normalized breast (*nD_breast_*) and lung (*nD_lung_*) doses were subsequently calculated. The differences between OBTCM and ATCM normalized organ dose estimates were tested using linear regression models that included CT scanner and *D_w_* as covariates.

**Results:**

Mean dose reduction from OBTCM in *nD_breast_* was significant after adjusting for the scanner models and patient size (*P* = 0.047). When pooled with females and male patient, mean dose reduction from OBTCM in *nD_lung_* was observed to be trending after adjusting for the scanner model and patient size (*P* = 0.085).

**Conclusions:**

One specific manufacturer’s OBTCM was analyzed. OBTCM was observed to significantly decrease normalized breast relative to a modeled version of that same manufacturer’s ATCM scheme. However, significant dose savings were not observed in lung dose over all. Results from this study support the use of OBTCM chest protocols for females only.

## 1 INTRODUCTION

Computed tomography (CT) was first introduced in the 1970s, and the technology has rapidly evolved making it an important and highly utilized diagnostic tool for clinicians. In 2007, an estimated 62 million CT exams were performed annually with an annual growth rate of 12.5% from 2008 to 2009.^1^, ^2^ The increased utilization of CT procedures, and particularly the frequency of exams per patient, has raised concern about potential carcinogenic health risk from the associated radiation, despite the fact that the potential cancer risk associated with a single CT scan is considered to be very low or non‐existent.^1^, ^3^ Another important aspect when considering radiation‐related carcinogenesis is the radiation dose to radiosensitive organs, such as the breast. The breast is one of the most radiosensitive organs and is incidentally irradiated during certain CT exams while not being the organ of interest, for example, when evaluating the lung for pulmonary embolism using a thoracic CT exam.

Several approaches have been explored to reduce radiation dose to the breast from CT procedures. For instance, radioprotective shields made with bismuth can attenuate the entrance exposure during the CT exam thereby reducing the breast dose.^4^, ^5^, ^6^ However, it has been demonstrated that these protective devices can negatively impact image quality by increasing noise, create streak and beam hardening artifacts, and affect CT number accuracy.^7^, ^8^, ^9^ CT technology advancements have widely replaced the use of bismuth breast shields by incorporating techniques such as automatic exposure control (AEC). One type of AEC which is used routinely in clinical practice is the attenuation‐based, automatic tube current modulation (ATCM). ATCM adjusts the x‐ray tube current along the angular and/or longitudinal direction to optimize the dose distribution based on the patient’s overall attenuation and provide overall improved image quality at a reduced radiation dose.^10^

A more recent evolution in CT technology provides additional radiation dose savings, particularly to radiosensitive organs, with organ‐based tube current modulation (OBTCM). This technique reduces the x‐ray tube current preferentially over projections containing the radiosensitive organ. For instance, the anterior (assuming the patient is positioned supine) x‐ray tube current is reduced in a chest CT to decrease dose to the breast. One OBTCM approach reduces the x‐ray tube current over the anterior 120° of the patient with the aim of redistributing the dose posteriorly to reduce dose to the anterior organs (including breast) while maintaining image quality.^11^, ^12^

Several investigators have quantified the anterior dose distribution from OBTCM relative to conventional ATCM using physical measurements with dosimeters in anthropomorphic phantoms. A study conducted by Lungren et al. used MOSFET detectors on an adult, female anthropomorphic thoracic phantom to measure the anterior dose distribution from OBTCM and reported a 17‐47% decrease anteriorly with accompanying maximum 52% increase posteriorly relative to ATCM.^13^ A similar study done by Matsubara et al. used radiophotoluminescence dosimeters to specifically investigate the breast dose with OBTCM and reported a 22% reduction.^14^

The use of Monte Carlo (MC) simulation techniques have also been used to quantify breast dose reduction from OBTCM. MC studies conducted by Fu et al. and Franck et al. have noted 15% and 11% breast dose reduction, respectively, from angular tube current reduction strategies.^15^, ^16^ Additionally, a few recent MC studies have noted an increase of lung dose with the use of OBTCM relative to conventional ATCM. Franck et al., for example, noted an average 11% increase in lung dose with OBTCM relative to conventional ATCM in female, oncologic patients with MC simulations from ImpactMC.^15^ Similarly, Lopez‐Rendon et al., observed an average 7.8% increase in lung dose when comparing OBTCM to conventional ATCM in female and male cadavers.^17^ Additionally, the MC studies conducted by Fu et al. using the female XCAT phantoms showed small decrease in lung dose when using a 180° of fluence reduction.^16^, ^18^ This can be confounding when considering both breast and lung tissues are equally radiosensitive per ICRP 103 tissue weighting recommendations.^19^ Although the use of OBTCM may spare dose to anteriorly located radiosensitive organs, the potential increased dose to posteriorly located organs may result in no net benefit in terms of overall population risk.^18^, ^20^

While phantom studies consider the effects of OBTCM on dose distribution, they have two important limitations in terms of evaluating breast dose. The first limitation is that phantom models are often not representative of actual patient anatomy since positioning of the breast may place the radiosensitive tissue within the area of increased radiation due simply to tissue movement or deformation. The study by Lungren et al. observed that for adult female patients, the average angle needed to contain all breast tissue was 155°.^13^ Moreover, other investigators have demonstrated, for actual adult female patients, the breasts may not be positioned within the OBTCM angular range of reduced fluence when the patient is in the supine position.^15^, ^21^, ^22^ The second limitation is that point dose measurements from dosimeters may not reflect the average dose to the organ of interest (e.g., the breast) due to the heterogeneity of the dose distribution within the patient (or more specifically the breast). This can be especially true when that distribution is not uniform and particularly near the surface,^23^ such as estimating dose to the breast from helical scans and especially when some form of tube current modulation (including angular) modulation is being used.

MC‐based simulation studies also possess some limitations. The MC approach employed by Franck et al. only had direct access to the actual z‐axis modulation of the tube current and therefore had to model the angular modulation of the tube current based on some approximations.^15^ In addition, their within‐patient comparison of OBTCM to ATCM involved using scans of oncology patients scanned 6 months apart. While this is a reasonable approximation, that approach did not account for any change in patient positioning, weight gain/loss or other issues between scans. Finally, their study did not consider the effects of patient size. Lopez‐Rendon et al. was able to more directly compare OBTCM and conventional ATCM due to the use of cadavers but was limited in terms of the number of patients.^17^ Both studies of Fu et al. utilized computational patients and theoretical expressions of OBTCM and ATCM that may not take into account limitations of clinical systems and thus used modulation functions that might not be realized in the clinical setting.^16^, ^18^ In addition, the use of only female models presents the same issue of not considering the clinical relevance of dose penalties or savings for males as mentioned above.

Therefore, the purpose of this work was to estimate and compare breast and lung doses of chest CT scans using OBTCM to estimates of ATCM for both female and male patients and across a range of patient sizes. To overcome the limitations of the previous work, a validated MC simulation approach was used to accurately model the CT scanner, the patient and actual organ‐based TCM schemes that were patient‐specific. In order to perform a direct within‐patient comparison of lung and breast dose from OBTCM relative to conventional TCM, a previously developed and validated method was used to model the conventional ATCM scheme based on CAREDose4D^24^ (Siemens Healthineers, Forchheim, Germany). This study considered organ doses from OBTCM relative to ATCM for females, males, and pooled populations, with the pooled being used to determine the effects of having one OBTCM protocol used for both females and males.

## 2 MATERIALS AND METHODS

This study employed MC simulation methods for CT radiation transport to compare lung and breast doses from clinical OBTCM scans to those of estimates of conventional ATCM. To obtain as realistic an estimate of organ dose as possible, actual tube current modulation data were extracted from clinical performed scans on individual patients (both male and female). Specifically, the OBTCM tube current information was extracted from the raw projection data collected from clinical patient scans obtained directly from the CT scanners. Additionally, the image data that resulted from the OBTCM scans were used to create voxelized patient models that were specific to the scan performed. Because the patients were only scanned once clinically, a direct comparison to ATCM was not possible, so the conventional ATCM tube current information was estimated using the attenuation information in the topogram as described by McMillan et al.^25^ Both of these TCM schemes were then incorporated separately into MC simulations on a per‐patient basis for a direct comparison between OBTCM and ATCM lung and breast dose. The details of this approach are provided below.

### 2.A Patient models

To estimate the effects of OBTCM relative to ATCM on breast and lung dose, image and raw projection data were collected under IRB approval (PA12‐0496) for 34 patients (17 males, 17 females) obtaining standard of care chest/abdomen/pelvis (CAP) CT examinations. For the 34 exams, 19 scans were obtained from the SOMATOM Force (VA50A, Siemens Healthineers, Forchheim, Germany) and 15 scans from the SOMATOM Definition Flash (VA48A, Siemens Healthineers, Forchheim, Germany). For this study, only the chest portion of the CAP exams was used. The chest portion of all CAP examinations used OBTCM and were acquired with the patient in the supine position. Additionally, all images were reconstructed to 500 mm full field‐of‐view (FOV) in order to ensure that the patient anatomy was contained within the image data. This includes the glandular breast tissue for female patients. The patient size in terms of water equivalent diameter (*D_w_*) was determined for the image data of each patient at the center of the volume.^26^ Specifically, *D_w_* was estimated using an ROI that encompassed the patient anatomy in the central cross‐sectional image.

**Fig. 1:**
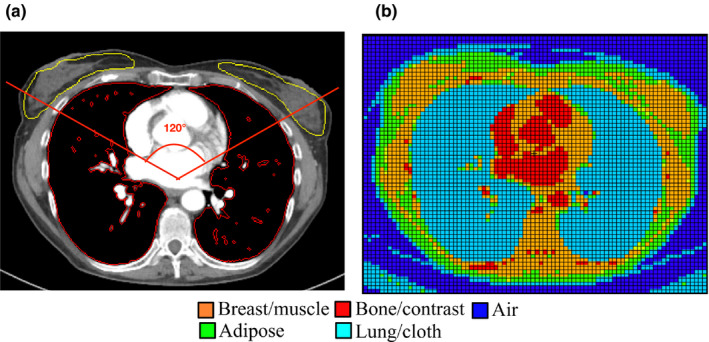
a) Segmented image and b) voxelized patient model of a female patient who underwent clinically indicated CAP CT exam for use in MC simulations. The segmented image contains an outline of 120*°* fluence reduction zone.

In order to use patient data in MC simulations, voxelized models of each patient’s anatomy were created from the image data. Voxels within each image series were modeled as either lung, fat, water, muscle, bone or air then subdivided into one of 17 density levels depending on their CT number.^27^ The lung and glandular breast tissues were semi‐automatically contoured and identified in the female models. Lung tissue was also segmented in male models, but glandular breast tissue was not in male patients.^28^ Figure 1 contains images of a female patient with lung and breast tissues segmented.

### 2.B CT scanning protocol

The CT chest protocols used to acquire the raw data from the two scanners are shown in Table 1. For 10 cases acquired on the Flash scanner, a tube voltage of 100 kVp was applied due to CARE kV (Siemens Healthineers, Forchheim, Germany) being utilized for these scans. For all 34 cases, scans were acquired using the OBTCM technique offered by the manufacturer (XCARE, Siemens Healthineers, Forchheim, Germany). This OBTCM algorithm employs an angle of 120° for fluence reduction that cannot be changed by the user. To provide comparisons with an ATCM acquisition on the same patients, the methods developed by McMillan et al.^25^ were applied to produce predicted tube current modulation profiles for each patient using the same scanner parameters shown in Table 1. Included in Table 1 are the software versions for the Force and Flash. The details of generating predicted ATCM schemes are based on these scanning parameters are briefly described below in Section 2. C.

**Table 1:** Scanning parameters used for the chest portion of the CAP exam for the Siemens Force and Flash scanners. The software versions for the Force and Flash scanners are provided.

Parameter	Siemens Force (VA50A)	Siemens Flash (VA48A)
kVp	120	120/100*
Quality reference mAs (QRM)	140	140/269*
Rotation time (s)	0.5	0.5
Pitch	0.6	0.6
Nominal collimation (mm)	57.6 (96 × 0.6 FFS^†^)	38.4 (64 × 0.6 FFS^†^)
Measured collimation (mm)	59.6	42.9
Bowtie filter	Body	Body
HVL (mm Al)	8.0	7.9/6.8*
CTDI_vol_ (mGy/mAs)	0.115	0.140/0.087*

* 10 patients on the Flash had scans performed with 100 kVp due to CARE kV and hence had a different QRM, HVL, and CTDI_vol_ per mAs value.

† Flying Focal Spot (FFS) uses periodic motion in the longitudinal (z‐axis) direction for double sampling.

### 2.C Modeling tube current modulation schemes

OBTCM schemes were extracted directly from raw projection data for each patient. The tube current, *I*, was expressed as a function of table position, and tube angle, *I*(*z, Θ*) where *z* represents the table position and *Θ* is the tube angle within the gantry.

The Siemens ATCM algorithm (CAREDose4D) utilizes both longitudinal and angular TCM. The longitudinal TCM accounts for the AP and LAT dimensions, yet only uses the maximum attenuation (*A_max_*) value in either the AP or LAT dimension for current software level. *A_max_* is calculated at each table position (*i*) based on the patient attenuation characteristics within the CT localizer radiograph (referred to by Siemens as the “topogram”) from [Eq. 1] as follows^25^:

(1) $$A_{\max}\left(i\right)=\max\left(\exp\left(\mu_{water,kVp}\times AP\left(i\right)\right),\exp\left(\mu_{water,kVp}\times LAT\left(i\right)\right)\right)$$

where *μ_water, kVp_* is the linear attenuation coefficient of water for a given beam energy. For this investigation, *μ_water, kVp_* was set to 0.2 cm^‐1^ for a 120 kVp beam. The maximum attenuation at each table position from Eq. 2 is then compared to a reference attenuation value (*A_ref_*) and used to calculate the longitudinal tube current (mA) at each table position, *i*:

(2) $$mA\left(i\right)=\left(\frac{QRM\times pitch}{t}\right)\times {\left(\frac{A_{\max}\left(i\right)}{A_{\mathrm{ref}}}\right)}^{b}$$

where QRM is the quality reference effective tube current‐product set by the user on the CT scanner, *t* is the gantry rotation time, *A_ref_* is the protocol‐specific reference attenuation hard coded into the ATCM algorithm, and *b* is a strength parameter set by the user to control the rate by which the tube current increases or decreases. In this study, the QRM was set to 140 based on the radiologist’s preference for the desired image quality of an average sized patient. The strength parameter (*b*) was set to “Average”, which corresponds to 0.33 for attenuation greater than *A_ref_* and 0.5 for attenuation less than *A_ref_*.^25^, ^29^ The angular current tube was calculated at each table position, *i*, for a helical scan using [Eq. (3)]

(3) $$m\left(i\right)=1‐\mu \left(i\right)\times \left(\frac{A_{\max}^{q}‐A{\left(i‐hROT\right)}^{q}}{A_{\max}^{q}‐A_{\min}^{q}}\right)$$

where *hROT* is the half rotation of the tube given by one half the collimation multiplied by the helical pitch, *A*(*i* – *hROT*) is the patient attenuation at the table position a half rotation prior to the current table position, *A_min_* is minimum patient attenuation over the previous half rotation, *A_max_* is the maximum patient attenuation over the previous half rotation, *q* is an optimization parameter between 0.5 and 1.0, and *μ*(*i*) is a gantry rotation time‐dependent parameter that limits the amount of modulation allowed at a given table position.^25^ The complete estimated ATCM schemes were calculated by multiplying longitudinal [from Eq. (2)] and the angular [from Eq. (3)] together.

**Fig. 2:**
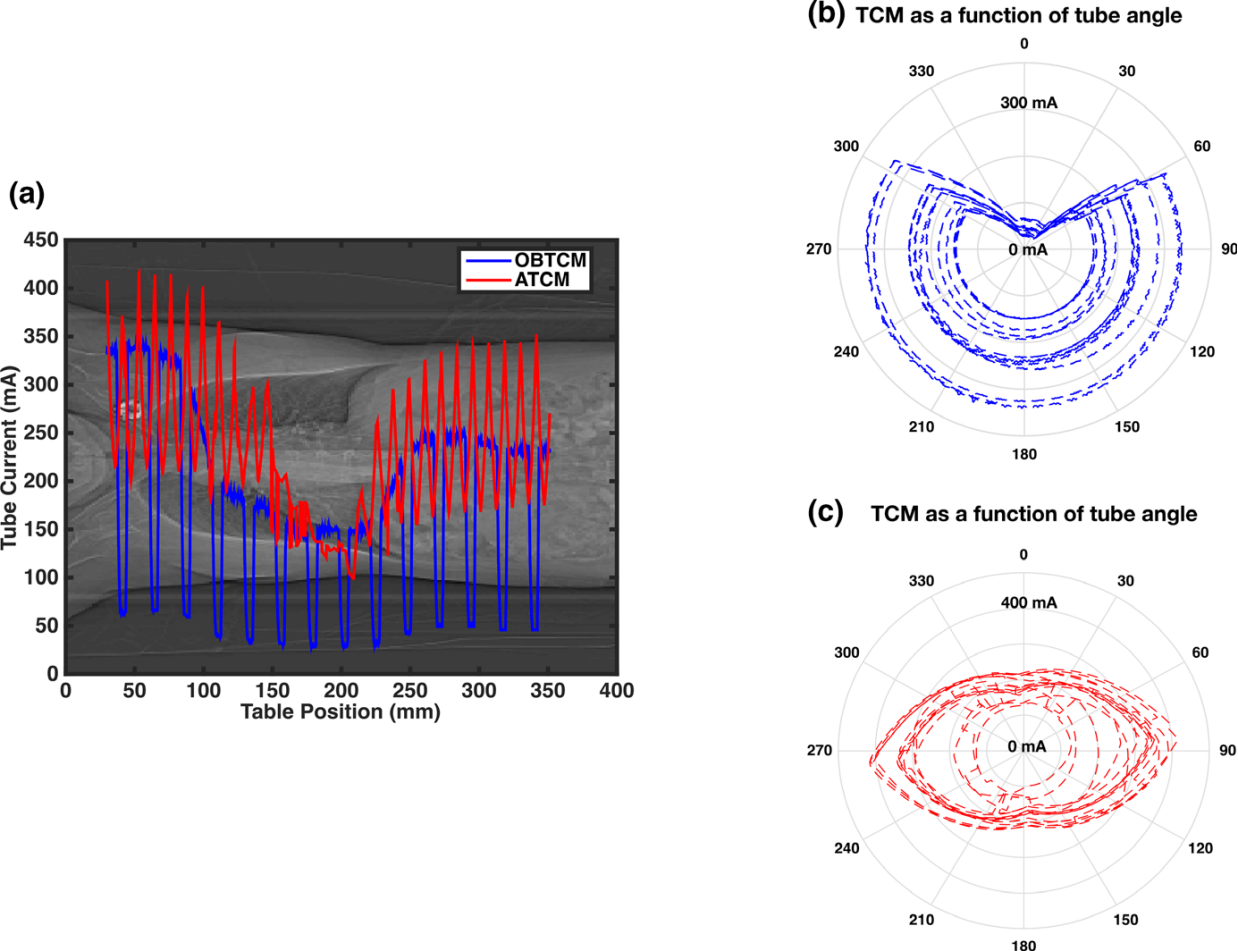
a) Extracted OBTCM (blue) and predicted ATCM (red) tube current schemes as a function of patient position overlaid atop of a patient topogram. Polar plots of b) OBTCM and c) ATCM as a function of tube gantry angle. For b) and c), the radial axes correspond to the tube current while the polar axes correspond to the gantry angle.

**Fig. 3:**
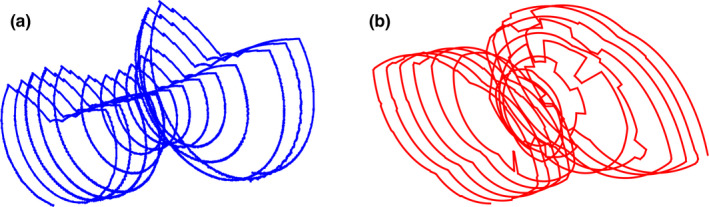
Three‐dimensional rendering of a) OBTCM and b) predicted ATCM tube current schemes. Note that in b), for OBTCM, the tube current is reduced on the anterior surface along the entire length of the scan and not just over the breast region.

Predicted ATCM schemes were generated for the table positions corresponding to the chest portion of the CAP topogram. In order to perform a direct per‐patient comparison with the OBTCM schemes extracted from the raw projection data, the ATCM schemes were estimated in accordance with the same imaging protocols specified in Section 2. B. Figure 2 depicts an extracted OBTCM scheme and predicted ATCM scheme of overlaid on a patient topogram. Figure 3 shows three‐dimensional renders of the OBTCM and ATCM tube current profile.

### 2.D MC simulations and dose calculations

A modified version of the MC software package MCNPX (Monte Carlo N‐Particle eXtended version 2.7.a) was utilized for all the simulations in this study.^30^, ^31^ The modification allowed for the modeling of MDCT scanner geometry and beam spectrum.^32^, ^33^, ^34^, ^35^ Specifically, in this investigation, the appropriate beam energy spectrum data were generated using the equivalent source method developed by Turner et al.^36^ An equivalent source model of either the Force or Flash scanners, as applicable, was used for each patient. Other user‐specified variables that define CT source trajectory such as scan start, scan location, and helical pitch were stipulated in the MCNPX input file. The effect of the Flying Focal Spot (FFS) was not considered in MC simulations. The effects are expected to be negligible in CT dosimetry because the use of the FFS does not increase the number of photons generated from the source, nor does it increase the fluence from the source. All simulations were conducted in photon transport mode with a 1 keV low‐energy cut‐off. This mode does not transport secondary electrons and assumes their energy to be deposited at the interaction site. Additionally, each simulation was performed with 10^7^ particle histories to ensure a statistical uncertainty of less than 1%. This MC simulation package has been validated under various conditions, including TCM.^37^, ^38^ MCNPX simulations were performed using the computational and storage services associated with the Hoffman2 Shared Cluster provided by UCLA Institute for Digital Research and Education’s Research Technology Group.

An additional text file containing the *I*(*z, Θ*) TCM information outlined in Section 2.C was utilized in the MC simulations. All values *I*(*z, Θ*) were normalized by the maximum tube current value then used as weighting factors at each angle (*z, Θ*) along the source trajectory in MC simulations. For the direct comparison, OBTCM and ATCM schemes were incorporated separately into MCNPX for each patient to get absolute lung and breast doses estimates for each modulation scenario.

### 2.E CTDI_vol_ values

For each patient, CTDI_vol_ for the chest portion of the CAP scan using OBTCM was taken from the patient protocol page. In order to estimate CTDI_vol_ for the predicted ATCM simulated scans, the collimation and bowtie‐specific CTDI_vol_ per mAs values given in Table 1 were multiplied by the average tube current‐time product across the entire simulated scan length. The CTDI_vol_ per mAs values were obtained from air kerma measurements performed on a 32‐cm CTDI phantom for both the Force and Flash at the appropriate tube voltages and normalized by the applied tube current‐time product. CTDI_vol_ values for OBTCM and estimated ATCM were correlated with *D_w_*. CTDI_vol_ values for OBTCM and ATCM were compared against one another in order to determine the relationship between the two. In both of these cases, the strength of the correlation was determined using coefficient of determination (*R^2^*).

### 2.F Dose analysis

Absolute breast and lung (*D_breast_* and *D_lung_*) doses tallied in Section 2.D for OBTCM and ATCM were normalized by the respective scan‐specific 32‐cm CTDI_vol_ values to yield normalized breast and lung doses (*nD_breast_* and *nD_lung_*). Comparisons between OBTCM and ATCM for normalized doses were done on a per‐patient basis by calculating within‐patient difference (%) relative to ATCM. Comparisons were done for: 1) *nD_breast_* and *nD_lung_* for females, 2) *nD_lung_* for males, and 3) *nD_lung_* for both females and males (pooled). Comparisons were conducted with CTDI_vol_‐normalized doses in order to account for the radiation output from two scanners and the different protocols used in this investigation.^39^ Negative differences were interpreted as dose savings relative to ATCM, while positive differences were interpreted as dose penalties relative to ATCM.

### 2.G Statistical analysis

Summary statistics for patient size and CTDI_vol_ values for OBTCM and ATCM were reported for females, males, and the pooled population. Coefficient of determination (*R^2^*) was used to assess the proportion of the variability observed in OBTCM CTDI_vol_ can be explained with the estimated ATCM CTDI_vol_. The mean, median, and standard deviation (SD) for Δ*nD_breast_* and Δ*nD_lung_* were also reported for females, males, and the pooled population. Differences for *nD_breast_* and *nD_lung_* relative to ATCM (Δ*nD_breast_* and Δ*nD_lung_*) for females and males were parameterized with respect to *D_w_*. The differences between OBTCM and ATCM in *nD_breast_* (for females) and pooled *nD_lung_* estimates were tested using linear regression models that included the CT scanner and *D_w_* as covariates.

As mentioned in Section 2C, this study utilized the methods of McMillan et al. to estimate ATCM in the absence of raw projection data. The McMillan et al. study compared MC‐derived organ doses from actual ATCM to the estimated ATCM (Table 3). In this study, tolerance limit intervals were employed to identify the extent of cases where the differences between organ doses from actual OBTCM and estimated ATCM exceeded the expected variability of organ doses from ATCM seen in McMillan et al. Specifically, the variability in organ dose data from the actual vs the estimated ATCM profiles was calculated from Table 3 of that study.^25^ A tolerance limit interval covering 90% of the population with 95% confidence level was then calculated with non‐central *t*‐distribution per organ for females, males, and the pooled population of females and males. After identifying the cases within and outside of the tolerance limit, one‐sample proportion tests were performed to determine whether the chance of occurring outside of the tolerance limit is random.^40^ All statistical analyses were performed in Stata (v. 14.1, College Station, Texas).

## 3 RESULTS

### 3.1 3. A Patient Size and CTDI_vol_ values

**Fig. 4:**
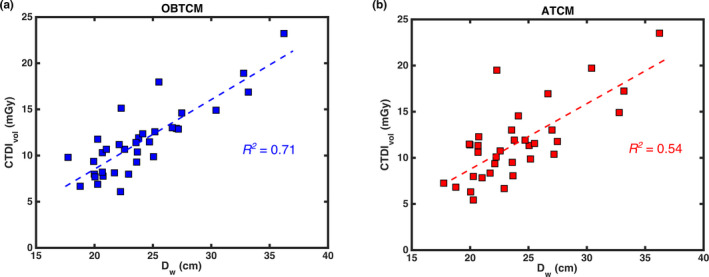
Correlation of a) OBTCM CTDI_vol_ and b) ATCM CTDI_vol_ with respect to *D_w_*.

**Fig. 5:**
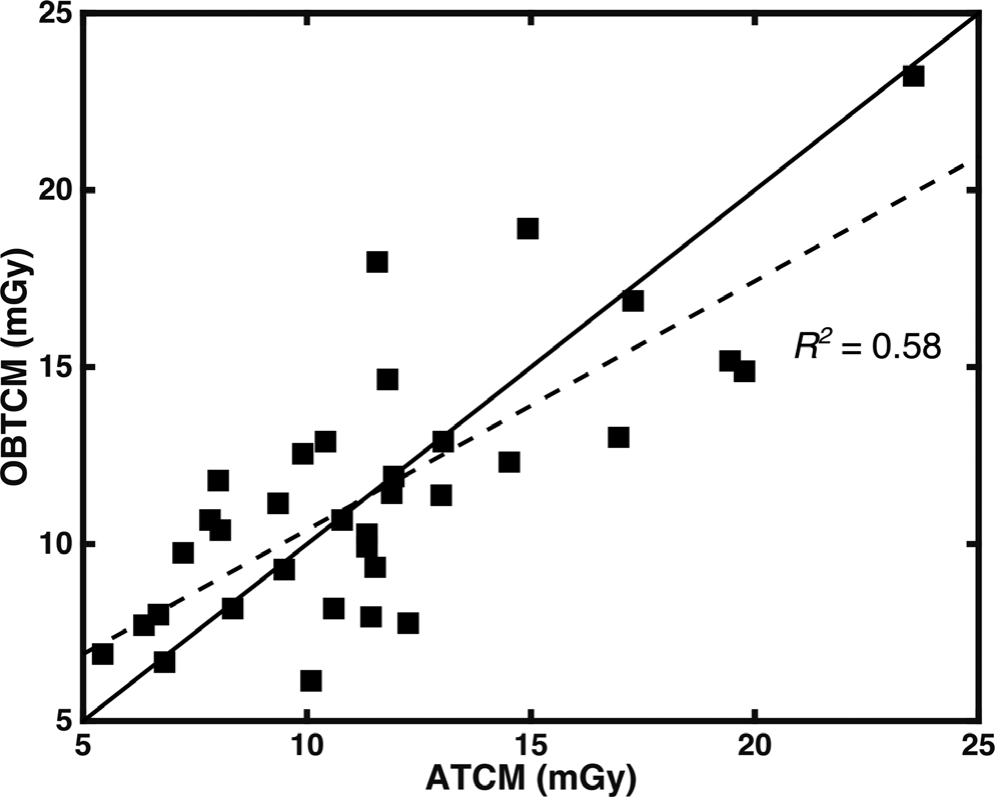
Plot of OBTCM CTDI_vol_ in relation to predicted ATCM CTDI_vol_. The dashed line represents the correlation between OBTCM and ATCM CTDI_vol_ of which the coefficient of determination (*R* ^2^) was observed to be 0.58. The solid line represents the line of identity (CTDI_vol_ from OBTCM equal to CTDI_vol_ from ATCM).

The measured range of *D_w_* was 20.0–33.2 cm for females. The mean, median, and standard deviation of *D_w_* for females was 23.5 cm, 22.9 cm, and 3.9 cm, respectively. The range of *D_w_* for males was 17.8–36.2 cm, with the mean, median, and standard deviation of *D_w_* being 24.3 cm, 23.6 cm, and 4.9 cm, respectively. For females, the CTDI_vol,32_ range for OBTCM was observed to be 6.1–16.9 mGy with the mean, median, and standard deviation being value 9.9 mGy, 9.3 mGy, 3.0 mGy, respectively, and the CTDI_vol_ range for simulated ATCM was observed to be 5.4–17.3 mGy with the mean, median, standard deviation 10.1 mGy, 10.4 mGy, and 3.0 mGy, respectively. For males, the CTDI_vol_ range for OBTCM was observed to be 8.2–23.2 mGy with the mean, median, and standard deviation being value 13.1 mGy, 11.8 mGy, 3.9 mGy, respectively, and the CTDI_vol,32_ range for simulated ATCM was observed to be 7.0–23.5 mGy with the mean, median, standard deviation 13.0 mGy, 11.6 mGy, and 4.6 mGy, respectively. Table 2 contains the summary statistics of *D_w_* and CTDI_vol_ values for females, males, and pooled females and males. Figure 4 shows the Pearson correlations of OBTCM and ATCM CTDI_vol_ values with respect to *D_w_* across all patients using a linear relationship. The *R^2^* values for OBTCM and ATCM with respect to *D_w_* were observed to be 0.71 and 0.54, respectively. The correlation of CTDI_vol_ between OBTCM and ATCM results is shown in Figure 5. The *R^2^* value between OBTCM and ATCM CTDI_vol_ was observed to be 0.58.

**Table 2:** Summary statistics of *D_w_* and CTDI_vol_ values for females, males, and for pooled females and males.

	*D_w_* (cm)			OBTCM CTDI_vol_ (mGy)			ATCM CTDI_vol_ (mGy)		
	Mean	Median	SD	Mean	Median	SD	Mean	Median	SD
Females	23.5	22.9	3.9	9.9	9.3	3.0	10.1	10.4	3.0
Males	24.3	23.6	4.9	13.1	11.8	3.9	13.0	11.6	4.6
Pooled	23.9	23.3	4.3	11.5	10.6	3.8	11.6	11.4	4.1

### 3.B Organ dose comparisons between OBTCM and ATCM

#### 3.B.1 Breast and lung dose comparison for females

For females, the OBTCM difference relative to ATCM for *nD_breast_* (Δ*nD_breast_*) was observed to range from −31% to 21%. The mean, median, and standard deviation (SD) of the difference from ATCM for females was −10%, −13%, and 16% for Δ*nD_breast_*, respectively. An *R^2^* value of 0.28 was observed when correlating Δ*nD_breast_* from OBTCM with *D_w_*. Mean dose reduction of OBTCM in *nD_breast_* was significant after adjusting for the scanner models and *D_w_* (*P* = 0.047). The *nD_lung_* for females demonstrated a difference relative to ATCM (Δ*nD_lung_*) ranging from −18% to 26%. The mean, median, and standard deviation of the difference from ATCM for females was −2%, −4%, and 12%, respectively, for Δ*nD_lung_*. The normalized dose in the Flash scanner model was observed to be significantly higher than the Force model with the mean of 10% and −7%, respectively. Tables 3 and 4 contains *nD_breast_* and *nD_lung_* comparisons, respectively.

**Table 3:** OBTCM and ATCM normalized breast dose (*nD_breast_*) comparison for females. Δ*nD_breast_* are reported as percentages relative to ATCM comparison.

ID	*D_w_* (cm)	OBTCM		ATCM		Δ*nD_breast_* (%)
		CTDI_vol_ (mGy)	*nD_breast_*	CTDI_vol_ (mGy)	*nD_breast_*	
Flash						
2*	20.0	8.0	0.84	11.4	1.05	−20
5*	20.7	7.8	0.79	12.3	0.76	4
6*	22.2	6.1	1.17	10.1	1.22	−5
8*	22.9	8.0	0.96	6.7	1.11	−13
11	25.1	9.9	0.87	11.4	0.95	−9
Mean						−11
Force						
16	18.8	6.6	1.15	6.8	0.96	21
18	20.0	7.7	0.80	6.3	0.81	−1
19	20.3	6.9	0.94	5.4	0.83	14
22	21.7	8.2	0.83	8.4	0.96	−14
24	22.6	10.7	0.88	10.8	1.01	−12
25	23.6	9.3	0.95	9.5	1.36	−31
26	23.7	10.4	0.93	8.0	0.83	12
27	23.8	11.9	0.70	11.9	1.00	−30
31	27.0	12.9	0.88	13.0	1.14	−23
32	27.2	12.9	0.70	10.4	0.89	−22
33	27.5	14.6	0.63	11.8	0.76	−17
34	33.2	16.9	0.73	17.3	0.96	−25
Mean						
**Across scanners**						
Mean	23.5	9.9	0.87	10.1	0.98	−10
Median	22.9	9.3	0.87	10.4	0.96	−13
SD	3.6	3.0	0.15	3.0	0.16	16

* Performed with CARE kV protocol.

**Table 4:** OBTCM and ATCM normalized lung dose (*nD_lung_*) comparison for females. Δ*nD_lung_* are reported as percentages relative to ATCM comparison.

ID	*D_w_* (cm)	OBTCM		ATCM		Δ*nD_lung_* (%)
		CTDI_vol_ (mGy)	*nD_lung_*	CTDI_vol_ (mGy)	*nD_lung_*	
Flash						
2*	20.0	8.0	1.14	11.4	1.19	−4
5*	20.7	7.8	1.03	12.3	0.92	12
6*	22.2	6.1	1.58	10.1	1.26	26
8*	22.9	8.0	1.17	6.7	1.12	5
11	25.1	9.9	1.20	11.4	1.09	11
Mean						10
Force						
16	18.8	6.6	1.35	6.8	1.33	1
18	20.0	7.7	1.25	6.3	1.18	6
19	20.3	6.9	1.37	5.4	1.29	6
22	21.7	8.2	1.17	8.4	1.31	−11
24	22.6	10.7	1.15	10.8	1.28	−10
25	23.6	9.3	1.25	9.5	1.47	−15
26	23.7	10.4	1.15	8.0	1.21	−5
27	23.8	11.9	1.03	11.9	1.26	−18
31	27.0	12.9	0.97	13.0	1.13	−15
32	27.2	12.9	1.02	10.8	1.04	−2
33	27.5	14.6	0.78	11.8	0.85	−9
34	33.2	16.9	0.97	17.3	1.05	−8
Mean						−7
**Across scanners**						
Mean	23.5	9.9	1.15	10.1	1.18	−2
Median	22.9	9.3	1.15	10.4	1.19	−4
SD	3.6	3.0	0.19	3.0	0.16	12

* Performed with CARE kV protocol.

The tolerance limit covering 90% of the population with 95% confidence was observed to be [1.31%, 12.43%] in breast dose for females. Out of the total 17, 15 females (88%) had larger differences than the tolerance limit (*N_outside_*) with 2 being within the tolerance limit (*N_within_*). The number of cases outside of the tolerance limit indicates that the dose reduction exceeds the tolerance limit of ATCM’s reproducibility (*P* = 0.0016). The tolerance limit covering 90% of the population with 95% confidence for lung dose was observed to be [2.29%, 12.26%]. In the lung, 12 females (*N_outside_* = 71%) had larger differences than the tolerance limits, indicating that the dose reduction exceeds the tolerance limit of ATCM’s reproducibility (*P* = 0.089).

#### 3.B.2 Lung dose comparison for males

For males, the OBTCM differences relative to ATCM for *nD_lung_* (Δ*nD_lung_*) ranged from −21% to 36%. The mean, median, and standard deviation (SD) of the difference compared to ATCM for males was 9%, 13%, and 16% for Δ*nD_lung_*, respectively. Table 5 contains the lung dose comparison for males. The normalized dose in the Flash scanner model was observed to be significantly higher than the Force model with the mean of 16% and 0%, respectively (*P* = 0.038). The tolerance limit covering 90% of the population with 95% confidence *was* observed to be [1.29, 11.04%] in lung dose for males. Fourteen males (*N_outside_* = 82%) had larger differences than each group’s tolerance limits (*p* = 0.0075).

**Table 5:** OBTCM and ATCM normalized lung (*nD_lung_*) comparison for males. Δ*nD_lung_* are reported as percentages relative to ATCM comparison.

ID	*D_w_* (cm)	OBTCM		ATCM		Δ*nD_lung_* (%)
		CTDI_vol_ (mGy)	*nD_lung_*	CTDI_vol_ (mGy)	*nD_lung_*	
Flash						
1*	19.9	9.3	1.29	11.5	1.14	13
3*	20.7	10.3	1.24	11.3	1.04	20
4*	20.7	8.2	1.20	10.6	0.94	28
7	22.3	15.2	1.26	19.5	0.99	26
9*	23.6	11.4	1.09	13.0	0.85	28
10	24.7	11.5	1.27	11.9	1.15	11
12*	26.6	13.0	0.99	17.0	0.87	14
13*	30.4	14.9	1.01	19.7	0.89	14
14	32.8	18.9	0.96	14.9	0.84	14
15	36.2	23.2	0.80	23.5	0.86	−6
Mean						16
Force						
17	17.8	9.8	1.05	7.2	1.01	4
20	20.3	11.8	1.06	8.0	1.05	1
21	21.0	10.7	1.12	7.9	1.04	7
23	22.1	11.2	0.95	9.3	1.04	−9
28	24.1	12.3	1.04	14.5	1.29	−19
29	25.2	12.6	1.26	9.9	0.93	36
30	25.5	18.0	0.70	11.6	0.89	−21
Mean						0
**Across scanners**						
Mean	24.3	13.1	1.08	13.0	0.99	9
Median	23.6	11.8	1.06	11.6	0.99	13
SD	4.9	3.9	0.17	4.6	0.12	16

* Performed with CARE kV protocol.

#### 3.B.3 Pooled breast and lung dose comparison

When both populations were pooled (i.e., females and males), the mean, median, and standard deviation of the difference for Δ*nD_lung_* was 4%, 5%, and 15%, respectively. When correlating Δ*nD_lung_* with respect to *D_w_*, pooled across females and males, an *R^2^* value of 0.48 was observed. When pooled, mean dose reduction from OBTCM in *nD_lung_* was observed to be trending after adjusting for the scanner model and patient size (*P* = 0.085). A summary of Δ*nD_breast_* and Δ*nD_lung_* for all patients is given in Table 6. The tolerance limits covering 90% of the population with 95% confidence were observed to be [2.04%, 6.80%] in lung dose for the pooled group. Using the pooled tolerance limit, 28 out of 34 (*N_outside_* = 82%) had larger differences between OBCTM and ATCM than ATCM’s reproducibility (*P* = 0.0002). A summary of Δ*nD_breast_* and Δ*nD_lung_* and tolerance limit coverage for all patients are given in Table 6 and Table 7, respectively.

**Table 6:** Summary of female, male, and pooled mean, median, and standard deviation (SD) of differences (%) for Δ*nD_breast_* and Δ*nD_lung_*. Negative and positive differences were interpreted as dose savings and dose penalties relative to ATCM, respectively.

	Δ*nD_breast_*			Δ*nD_lung_*		
	Mean (%)	Median (%)	SD (%)	Mean (%)	Median (%)	SD (%)
Females	−10	−13	16	−2	−4	12
Males	—	—	—	9	13	16
Pooled	—	—	—	4	5	15

**Table 7:** Summary of female, male, and pooled tolerance limit coverage.

	Δ*nD_breast_*			Δ*nD_lung_*		
	*N_within_* (%)	*N_outside_* (%)	*N_total_*	*N_within_* (%)	*N_outside_* (%)	*N_total_*
Females	2 (12%)	15^+^ (88%)	17	5 (29%)	12 (71%)	17
Males	—	—	—	3 (18%)	14^+^ (82%)	17
Pooled*	—	—	—	6 (18%)	28^+^ (82%)	34

* Tolerance bound for the pooled statistics from the reference is narrower than by female and male.

^+^ Significantly different from 50% using proportional tests.

## 4 DISCUSSION

The objective of the introduction of OBTCM was to reduce the radiation dose to radiosensitive organs, particularly more anteriorly positioned ones. This study focused on the impact of this technique on organ dose, specifically investigating the potential radiation dose reduction or penalty to the breast in female patients and lung in male and female patients from an OBTCM chest exam relative to an ATCM chest exam that is typically used in the clinical setting. This comparison was achieved by extracting OBTCM tube current information directly from the raw projection data, estimating ATCM tube current data using the attenuation information found in the topogram, and incorporating both TCM schemes separately into MC simulations. The intra‐patient differences in both absolute and CTDI_vol_‐normalized breast and lung dose from OBTCM relative to ATCM were investigated across patient size. This study overcame the limitations of previous studies by: (a) using actual OBTCM curves extracted from the raw projection data and used directly in the MC simulation models so that the organ dose estimates were patient‐specific; (b) employing a previously published and validated method to estimate the conventional ATCM curves for each patient; (c) comparing directly the effects of OBTCM vs ATCM in terms of both lung and breast dose for both males and females; and (d) taking into account the effects of patient size.

In terms of CTDI_vol_, the *R^2^* values between CTDI_vol_ and *D_w_* for OBTCM and ATCM were 0.71 and 0.54, respectively. This suggests the *D_w_* explains 71% of variation of CTDI_vol_ from OBTCM but only 54% of the variation from ATCM. The decreased strength of the correlation of CTDI_vol_ with respect to ATCM could be due to the fact the tube current values are estimated. The relationship between OBTCM and ATCM, as indicated by the Pearson’s correlation coefficient shown in Fig. 5, was observed to be 0.58. This relationship suggests that, for a given protocol and for a given patient, the CTDI_vol_ values between OBTCM and ATCM may not necessarily be one‐to‐one. It is not known whether the OBTCM was designed to preserve the CTDI_vol_ for a given protocol and for a given patient in relation to ATCM. To maintain image quality with OBTCM, the posterior radiation dose must be increased for all projection angles except 120° anteriorly. The effect this posterior increase has on CTDI_vol_ in relation to ATCM for patients is, however, beyond the scope of this investigation. The irregularities and spikes (particularly in the shoulder region) seen in [Fig. 3(b)] may not appear in an actual ATCM scheme and are the results of the ATCM estimation. In terms of estimating organ dose, these irregularities only exist across a few projections relative to the entirety of the ATCM scheme and therefore would not drastically affect the breast dose estimates. Again, though, it should be noted that the ATCM schemes—and hence the CTDI_vol_—were estimated and could therefore be the source of the variation. Additionally, *D_w_* as a function of table position for chest scans can vary along the length of the patient. This study only looked at *D_w_* at the central slice as a metric for patient size, which could affect the strength of the correlations.

**Fig. 6:**
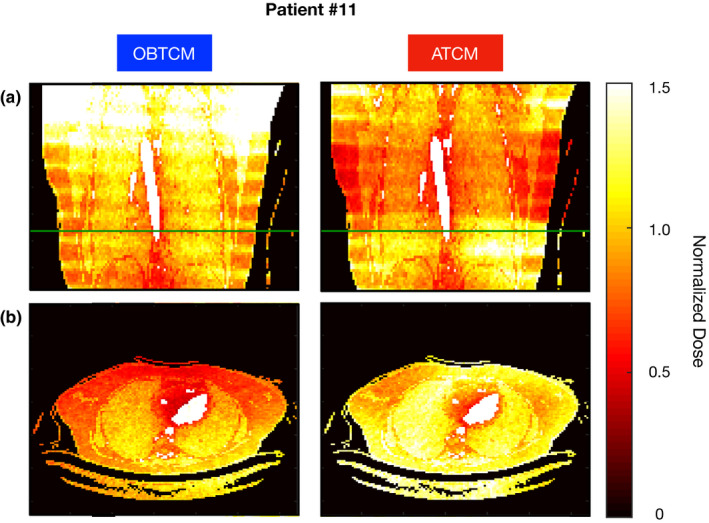
a) Coronal and b) axial dose distribution for patient #11 which had similar CTDI_vol_ values for the OBTCM (9.9 mGy) and estimated ATCM (11.4 mGy) exams. For this patient, there was a 9% breast dose saving but an 11% lung dose penalty. The green line in the coronal view represents the position where the axial slice was taken.

This study noted a significant mean dose reduction in *nD_breast_* from OBTCM after adjusting for the scanner models and patient size (*P* = 0.047). Furthermore, the results in Table 3 demonstrates an overall average reduction in the normalized breast dose (Δ*nD_breast_* = −10%) when using OBTCM compared to ATCM. For this study, male breast dose was not considered due to the absence of glandular tissue in the cross‐sectional images of the male patients. Additionally, the cancer biology of male breast cancer appears to be distinct from that of female breast cancer, both of which being outside the scope of this study.^41^ As can be seen in Table 3, there can be an increase in the normalized breast dose for some patients as the dose savings for Δ*nD_breast_* ranges from −31% to 21%. These variations in Δ*nD_breast_* are complex and based on several factors. For a specific patient, the increase or decrease in normalized breast dose for OBTCM relative to ATCM can be due to: (a) the degree and location of tube current reduction over the anterior portion of the chest (both OBTCM and ATCM do this), (b) the degree and location of tube current reduction over the longitudinal extent of the patient (again, both OBTCM and ATCM do this), and (c) exactly where the glandular breast tissue is positioned with respect to the gantry and whether all glandular tissue is contained within the 120° fluence reduction zone used by this implementation of the OBTCM. Similar to previous studies investigating OBTCM and breasting positioning, all of the females in the study possessed at least some breast tissue outside of the 120° fluence reduction zone.^15^, ^21^, ^22^ Dose maps for patient #11 are shown in Fig. 6 highlighting these complexities. Viewed in this light, the results suggest that the potential for breast dose reduction from OBTCM may be affected by the extent to which the breasts are within 120° fluence reduction zone. The tendency for the breasts to be displaced laterally while in the supine position can be mitigated with the use of brassieres, as their usage has been shown to position breast tissue more medially (and thus increase the proportion of breast tissue within the fluence reduction zone) without introducing image artifacts.^42^ However, as noted above, breast dose is also dependent on the longitudinal positioning of the breast tissue. Future work would necessitate a study OBTCM compared to ATCM as it relates to lateral and longitudinal breast positioning. These results highlight the strength of the MC simulation approach to this particular question of breast dose using OBTCM, as the use of idealized, rigid, physical phantoms to measure dose distributions simply does not take into consideration the variation of human anatomy nor tissue deformation when human anatomy is placed in certain positions.

Females also were observed to experience a small reduction, on average, in normalized lung dose with OBTCM relative to ATCM (Δ*nD_lung_* = −2%). Similar to the breast dose results, shown in Table 4, the observed range of normalized lung dose difference values (Δ*nD_lung_*: −18% to 26%) indicates some female patients experience dose penalties relative to ATCM. As noted previously, given OBTCM requires a posterior increase in tube current to maintain image quality, increased normalized lung dose would be expected for some patients. In contrast, males, on average, experienced an overall increase in normalized lung dose (Δ*nD_lung_* = 9%). However, as can be seen in Table 5, the range of observed differences of normalized lung dose (Δ*nD_lung_*: −19% to 36%) for males demonstrates some patients received a reduction in lung dose from OBTCM relative to ATCM. Despite this, when pooled across females and males, no significant difference in *nD_lung_* was observed after adjusting the scanner effect and patient size (*P* = 0.085). As noted previously, both breast and lung tissues are considered equally radiosensitive per ICRP 103 (*w_T_* = 0.12)^19^ and require equal consideration when utilizing OBTCM. Therefore, when the OBTCM chest protocol is applied to both females and males, the results of this study suggest that breast dose savings for women and lung dose penalties for men may not yield an overall benefit relative to ATCM in terms of effective dose, especially since effective dose is sex‐averaged.^19^ The results of this study do support the use of OBTCM chest protocols for female patients but not male patients. This being said, the data for Δ*nD_lung_* are trending toward reducing the mean dose. A larger patient pool might support the use of OBTCM for both females and males.

The MC study conducted by Franck et al. in 2018 showed a 9% reduction in breast dose.^15^ This result is comparable to the 10% dose reduction observed in this study. It should be noted that the Franck et al. study and this study used the same angular tube current reduction zone of 120^°^. This study, however, noted a small dose reduction in lung for female patients (Δ*nD_lung_* = −2%), as opposed to the 11% increase in lung dose for female patients seen in the Franck et al. study.^15^ The study performed by Fu et al. showed a 15 ± 2% reduction of normalized breast dose with the usage of 180^°^ reduction zone (referred to as organ dose‐based tube current modulation, ODM GE Healthcare).^16^ As such, increasing the angle of the tube current reduction zone to 180^°^ may provide greater dose reduction in terms of normalized breast dose relative to 120^°^. Additionally, the Fu et al. study also observed a small decrease in lung dose for female patients,^16^ as was seen in this study. However, the Franck et al. and Fu et al. studies did not investigate potential lung dose penalties for men when angular tube current reduction strategies are applied to both women and men. This current study observed a minor lung dose savings for women, but did observe lung dose penalties for men (Δ*nD_lung_* = 9%) when using the 120° tube current reduction zone. Results from this study demonstrate that investigating penalties and savings for both women and men with respect to different organ‐based modulation algorithms are warranted, especially since most facilities do not implement gender‐specific protocols.

This study has a few differences from previous work, particularly with respect to TCM data and patient size. The data presented herein are from patient models derived from clinical image data and actual OTBCM schemes extracted directly from the corresponding raw projection data. Additionally, the ATCM estimations employed herein are approximations of the methods of one manufacturer. As such, the data are not idealized but rather is reflective of real clinical scenarios.

This investigation does have some limitations. The first of these limitations is inclusion of only Siemens scanners and the OBTCM and ATCM AEC algorithms of Siemens. A more comprehensive study of breast and lung dose from OBTCM and ATCM should include other scanners and AEC algorithms from different manufacturers (such as ODM). Another limitation to this study is related to the heterogeneity of protocols coming from two scanners with some exams being performed with CARE kV. Ideally, a study of this nature would use a patient population from one scanner using one OBTCM and ATCM protocol for a direct, per‐patient comparison. This study attempted to assuage the effects of protocol heterogeneity with intra‐patient comparisons of absolute dose from OBTCM and ATCM. In addition, the effect of the scanner and patient size were used as covariates in linear regression in the comparison of dose differences. The mapping of material designations is performed based on CT numbers, so some of the spongiosa in the vertebral column got mapped as breast/muscle tissue (Fig. 1). This was not corrected in the simulation because it would require identifying all of the relevant voxels and changing their material (tissue) types. However, the effects in terms of lung and breast from this mischaracterization is expected to be minimal because the TCM schemes (both ATCM and OBTCM) are based on the attenuation information in the topogram, rather than on the exact tissue definition. The limitation of this study was in sample size of 17 per group. Considering the magnitude of ATCM, we tested the number of cases that had the greater magnitude of differences in dose between OBTCM and ATCM. A sample size of 17 in this study had approximately 80% power to show at least a proportion of 0.82 the differences in doses compared with random chance (i.e., 0.50) with two‐sided one‐sample proportional *z*‐test. This study used estimated ATCM as opposed to an actual ATCM directly from the raw projection data to make comparisons with OBTCM. This estimated ATCM does introduce around 5% error in terms of dose estimates.^25^ A comparison study of this nature would also ideally compare doses from ATCM values extracted from raw projection data. However, this was not an option because obtaining IRB approval for duplicate chest scans for this purpose alone was not considered feasible. This study did not investigate the effects of OBTCM on image quality relative to ATCM. OBTCM has been shown to increase image noise in relation to ATCM.^43^, ^44^ Lastly, this study did not consider the impact of patient centering as this study attempted to look at the best‐case scenario, which entailed the patient being centered in the gantry. Patient mis‐centering has been shown to yield consequences for the anterior‐posterior dose profiles, thereby affecting patient dose.^45^, ^46^

## 5 CONCLUSION

In this study, the OBTCM algorithm of one manufacturer (Siemens) was analyzed in relation to an estimated version of the ATCM algorithm of the same manufacturer. The OBTCM algorithm investigated in this study proports to reduce dose to anteriorly located organs such as the breast.^11^ This study found, on average, a reduction of normalized breast dose was observed with OBTCM relative to ATCM. However, the potential breast dose savings for females inherently comes at the expense of a small increase in normalized lung doses to both women and men. Results from this study support the use of OBTCM chest exams for females only. Overall, the results of this study highlight potential dose savings or penalties are variable and dependent upon patient size and breast positioning. Future work on this topic would examine the relationship of patient size on breast and lung doses, along with the impact of breast positioning specifically on breast dose with the use of OBTCM for human patients. Lastly, this study only investigated the fluence reduction angle implemented by one manufacturer. Future work would investigate the effects of different fluence reduction angles on lung and breast dose.

## Disclosures

M McNitt‐Gray: Departmental master research agreement, Siemens Healthineers, Forchheim, Germany; Research grant support, Siemens Healthineers, Forchheim, Germany; Member, Scientific Advisory Board, Hura Imaging, LLC, Los Angeles, CA. R Layman: Research agreement, Siemens Healthineers, Forchheim, Germany; research support from National Aeronautics and Space Administration and United States Department of Agriculture, Houston, TX.
